# Pulmonary Injury in Acute Experimental Pancreatitis Correlates With Elevated Levels of Free Fatty Acids in Rats

**DOI:** 10.1155/1992/92916

**Published:** 1992

**Authors:** H. R. Rosen, H. Tüchler

**Affiliations:** 1 Hanusch Medical Center Department of Surgery Heinrich Collinstr. 30 Vienna 1140 Austria; 2 Ludwig Boltzmann Research Institute Hanusch Medical Center Vienna Austria

## Abstract

Since some authors have stated a certain role for so-called “free fatty acids” (FFA) in the pathogenesis of AP and the subsequent systemic complications we tried to find possible correlations between FFA, pancreatitis and lung injury using a rat model. AP was induced by intraductal infusion of two different concentrations of glycodeoxycholic acid (GDOC 17 mmol and 34 mmol). An equal number of animals had only cannulation of the pancreatic duct without infusion and served as controls (GDOC-control). In another experimental model iv.-infusion of oleic acid (OA) was used to create severe lung injury comparable to human ARDS. In this model control animals received iv.-infusion of saline solution only (SAL). At 2,6,12,24 and 48 hours the animals were sacrificed and blood was collected for determination of FFA, amylase and pO2. The pancreas and lungs were removed for histologic examination and the lungs were weighed. GDOC-34 animals developed severe pancreatitis with hemorrhage and necrosis. Histology of the lungs showed edema, inflammatory infiltrates, hemorrhage and thickening of the alveolar membrane in GDOC-34 rats as well as in OA-animals. In contrast, there was only pancreatic edema until 24 hours in the GDOC 17 group and less severe histological changes in the lungs. Amylase, FFA, pO2 and lung weight were directly influenced by the different kinds of treatment. Furthermore, FFA correlated positively with the levels of amylase and lung weight and negatively with pO2. Infusion of OA alone also caused an increase in levels of amylase with pancreatic edema and focal necroses in some animals. These results show that it was possible to create different degrees of severity of AP which was in concordance with different degrees of morphologic changes and dysfunction in the lungs. FFA values correlated significantly with the clinical course as well as with increasing amylase, lung weight and decreasing pO2.


*HPB Surgery*, 1992, Vol. 6, pp. 79–90
Reprints available directly from the publisher
Photocopying permitted by license only

© Harwood Academic Publishers GmbH
Printed in the United States of America

### PULMONARY INJURY IN ACUTE EXPERIMENTAL PANCREATITIS CORRELATES WITH ELEVATED LEVELS OF FREE FATTY ACIDS IN RATS

H.R. ROSEN* and H. TÜCHLER**

**Hanusch Medical Center, Department of Surgery, Vienna, Austria*

***Ludwig Boltzmann Research Institute, Hanusch Medical Center, Vienna, Austria*

*(Received 24 February 1992)*

Since some authors have stated a certain role for so-called "free fatty acids" (FFA) in the pathogenesis of AP and the subsequent systemic complications we tried to find possible correlations between FFA, pancreatitis and lung injury using a rat model. AP was induced by intraductal infusion of two different concentrations of glycodeoxycholic acid (GDOC 17 mmol and 34 mmol). An equal number of animals had only cannulation of the pancreatic duct without infusion and served as controls (GDOC-control). In another experimental model iv.-infusion of oleic acid (OA) was used to create severe lung injury comparable to human ARDS. In this model control animals received iv.-infusion of saline solution only (SAL). At 2,6,12,24 and 48 hours the animals were sacrificed and blood was collected for determination of FFA, amylase and pO2. The pancreas and lungs were removed for histologic examination and the lungs were weighed. GDOC-34 animals developed severe pancreatitis with hemorrhage and necrosis. Histology of the lungs showed edema, inflammatory infiltrates, hemorrhage and thickening of the alveolar membrane in GDOC-34 rats as well as in OA-animals. In contrast, there was only pancreatic edema until 24 hours in the GDOC 17 group and less severe histological changes in the lungs. Amylase, FFA, pO2 and lung weight were directly influenced by the different kinds of treatment. Furthermore, FFA correlated positively with the levels of amylase and lung weight and negatively with pO2. Infusion of OA alone also caused an increase in levels of amylase with pancreatic edema and focal necroses in some animals. These results show that it was possible to create different degrees of severity of AP which was in concordance with different degrees of morphologic changes and dysfunction in the lungs. FFA values correlated significantly with the clinical course as well as with increasing amylase, lung weight and decreasing pO2.

KEY WORDS: Acute pancreatitis, glycodeoxycholic acid, rats, lung injury, free fatty acids

### INTRODUCTION

The incidence of pulmonary complications associated with acute pancreatitis has been reported to range from 15–70%[1–3]. Especially with hemorrhagic necrotizing pancreatitis a third of all patients are threatened by respiratory insufficiency that will require intensive care and mechanical ventilation. Despite this, up to 20% of all patients die of respiratory failure[4,5]. In a large series reported by Hollender and coworkers, 19% of patients died of respiratory insufficiency within the first 3 days after onset of the disease[6].

Address correspondence to: Harald R. Rosen M.D., Hanusch Medical Center, Department of Surgery, Heinrich Collinstr. 30, Vienna, Austria 1140

No definitive conclusions have yet been reached regarding the pathogenesis, pathophysiology and, in particular, the therapy of pancreatitis-induced lung injury. At present, explanations for the development of lung injury are largely speculative and inconclusive.

Some authors have postulated that lysolecithin and nonesterified fatty acids, so-called "free fatty acids" (FFA)[7–10] may play a role in the pathogenesis of the lung injury accompanying acute pancreatitis. Furthermore, a close correlation between the levels of FFA and the clinical outcome of patients with acute pancreatitis was recently described[7]. Therefore, we tried to correlate the time course of the morphologic changes of pancreas and lung with serum levels of FFA, arterial pO2 and amylase in a rat model of acute pancreatitis and in experimental ARDS.

### MATERIAL AND METHODS

This animal experiment was approved by the Animal Experimentation Committee of the Massachusetts General Hospital, Boston, Ma. Male Sprague-Dawley rats (Charles River) with a body weight of 350 g were used. The animals were fasted over-night prior to the experiment and had free access to water. Anesthesia was by 0.15 ml ketamine and 0.05 ml pentobarbital intraperitoneally. The carotid artery and jugular vein were cannulated with 25-gauge polyvinyl catheters. Through a mid-line laparotomy a 24-gauge polyethylene catheter was placed into the antimesenteric aspect of the duodenum opposite the papilla of Vater. The stylet was withdrawn and the catheter was advanced through the papilla into the distal common bile duct. An atraumatic clamp was placed on the proximal common bile duct just below the liver.

Pancreatitis was induced by infusion of 0.2 ml of either 17 mmol/l (*GDOC-17*) or 34 mmol/l (*GDOC-34*) glycodeoxycholic acid (GDOC) (Sigma Chemical Corporation, Saint Louis, MO, number G-325, sodium salt, molecular weight MW = 471.6 g/mol) into the bile duct cannula. Duct infusion was delivered by a pump-driven syringe over 2 minutes as described previously by Terry and coworkers, they reported a mean infusion pressure of 56 ± 10.4 cm $H_2O$[11]. The catheter was then removed and the duodenotomy was closed with a 6–0 prolene suture. Two minutes after injection the clamp was removed from the proximal common bile duct and the abdomen was closed with a running 4–0 suture. Animals in the pancreatitis control group (*GDDC-control*) had only cannulation of the pancreatic duct without infusion.

An equal number of animals had carotid artery and jugular venous catheters placed one day prior to the experiment. On the day of the experiment 0.05 ml of an isotonic oleic acid solution (= cis-9-octadecanoic acid; No. 0–3879, Sigma Chemical Co., St. Louis, MO.) was infused through the jugular venous catheter (*OA*). Another group of rats which received only the same amount of saline solution served as a control group (*SAL*).

After awakening, animals were kept in solitary cages with free access to water and rat chow. No further anesthesia was applied during the following observations. Since the last timepoint for evaluation was only 48 hours after induction of pancreatitis (Table 1) the use of antibiotics to prevent infection in the GDOC-17 or GDOC-34 animals was regarded as unnecessary. Previously published data describes the development of infection in acute experimental pancreatitis as a later event[12].

At predetermined timepoints (Table 1) animals were sacrificed by intravenous administration of 0.3 ml pentobarbital followed by rapid exsanguination via the carotid artery catheter. Blood was collected for determination of amylase, arterial pO2 and non-esterified free fatty acids. For determination of amylase a saccharogenic assay was used[13], measurement of FFA was performed by a colorimetic method using the WAKO TM NEFA C-test kit (WAKO Ltd., Japan) as described elsewhere[14].

**Table 1**

| *Time of sacrifice (hours after induction)* | *No of animals* | *Group* |
|---|---|---|
| 2 | 6 | GDOC 17 |
| 2 | 6 | GDOC 34 |
| 2 | 6 | GDOC-control |
| 2 | 6 | oleic acid (OA) |
| 2 | 6 | saline (SAL) |
| 6 | 6 | GDOC 17 |
| 6 | 6 | GDOC 34 |
| 6 | 6 | GDOC-control |
| 6 | 6 | OA |
| 6 | 6 | SAL |
| 12 | 6 | GDOC 17 |
| 12 | 6 | GDOC 34 |
| 12 | 6 | GDOC-control |
| 12 | 6 | OA |
| 12 | 6 | SAL |
| 24 | 6 | GDOC 17 |
| 24 | 6 | GDOC 34 |
| 24 | 6 | GDOC-control |
| 24 | 6 | OA |
| 24 | 6 | SAL |
| 48 | 6 | GDOC 17 |
| 48 | 6 | GDOC 34 |
| 48 | 6 | GDOC-control |
| 48 | 6 | OA |
| 48 | 6 | SAL |

Pancreas and lungs were removed immediately after exsanguination and the wet weight of the lungs was determined. Since the body weight was equal in animals (350 g) the determination of the wet lung weight was regarded as appropriate. Lungs and pancreas were placed in formalin for subsequent histologic examination.

The assignment of animals to the various groups was randomized.

*Statistical Methods*

The following statistical methods were used:

*a. Descriptive analysis*
Median values and ranges

*b. Effect of the experiment on the investigated parameters*
Kruskal-Wallis One Way Analysis of Variance

*c. Effect of experiment and/or time course on the investigated parameters*
Two Way Analysis of Variance (2-way ANOVA)

*d. Intercorrelation between all investigated parameters*
Kendall's Tau B

A $p$-value $<0.05$ was taken as the level of significance.

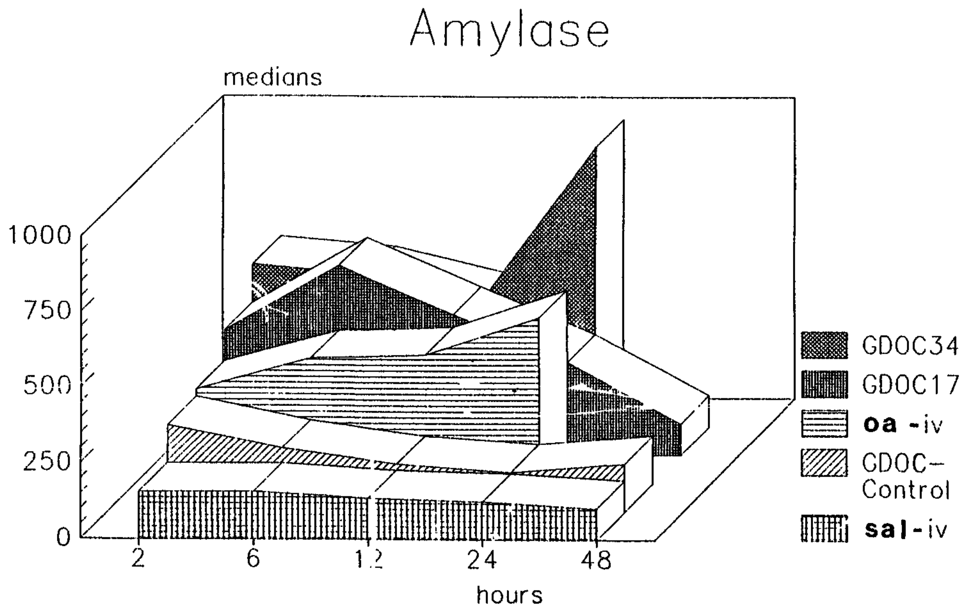

**Figure 1** Levels of Amylase (units); median values + (range)

| | 2 hrs | 6 hrs | 12 hrs | 24 hrs | 48 hrs |
|---|---|---|---|---|---|
| GDOC-34 | 535.5 (513.0) | 504.0 (290.0) | 413.5 (708.0) | 926.0 (853.0) | — |
| GDOC-17 | 408.0 (109.5) | 626.5 (403.0) | 463.0 (102.8) | 303.0 (353.0) | 103.5 (136.0) |
| GDOC-control | 281.0 (155.0) | 206.0 (186.0) | 155.0 (181.0) | 129.0 (140.0) | 157.0 (175.0) |
| OA i.v. | 305.0 (534.0) | 409.5 (451.0) | 420.0 (375.0) | 545.0 (235.0) | — |
| SAL i.v. | 155.5 (190.0) | 156.0 ( 85.0) | 135.0 (200.0) | 125.5 (153.0) | 102.0 ( 49.0) |

### RESULTS

The development of the four examined parameters during the experiment is illustrated in Figures 1–4. Since no animal in the GDOC-34 and in the OA-group survived longer than 30 hours the timepoint 48 hours was not evaluated.

Analysis of the effect GDOC-34, GDOC-17, OA, GDOC-control and SAL on the different variables by use of the one-way ANOVA test revealed a statistically significant impact of the different groups on all investigated parameters (Table 2).

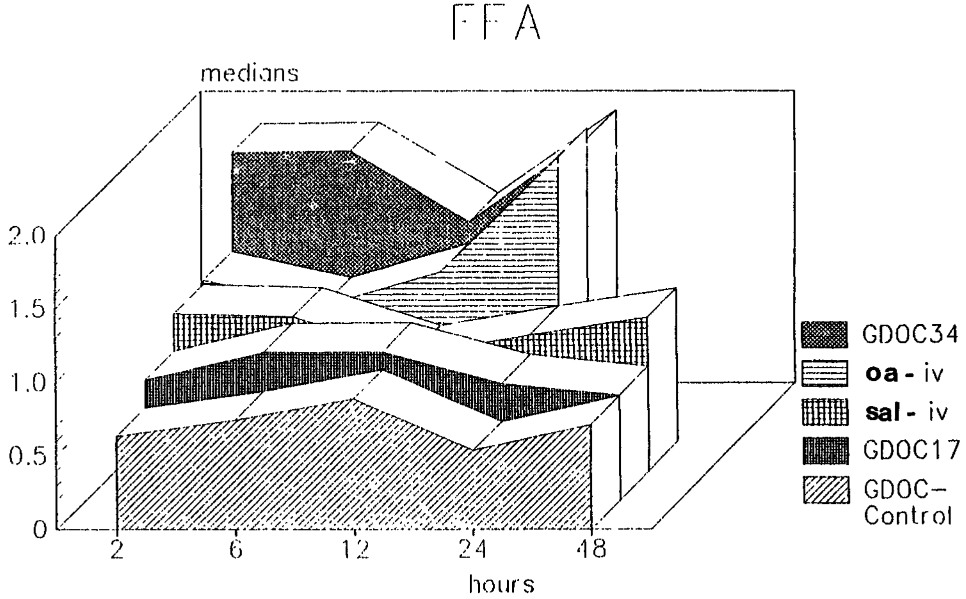

**Figure 2** Levels of FFA (mEq/l); median values + (range)

| | 2 hrs | 6 hrs | 12 hrs | 24 hrs | 48 hrs |
|---|---|---|---|---|---|
| GDOC-34 | 1.778 | 1.787 | 1.298 | 1.869 | — |
| | (2.893) | (1.365) | (0.362) | (0.829) | |
| GDOC-17 | 0.816 | 1.003 | 1.006 | 0.791 | 0.708 |
| | (0.972) | (1.183) | (0.307) | (1.190) | (0.620) |
| GDOC-control | 0.630 | 0.738 | 0.886 | 0.535 | 0.708 |
| | (1.075) | (0.694) | (.848) | (1.177) | (0.330) |
| OA i.v. | 1.095 | 0.917 | 1.152 | 1.940 | — |
| | (1.003) | (1.31]8) | (1.149) | (0.960) | |
| SAL i.v. | 1.066 | 1.046 | 0.790 | 0.921 | 1.038 |
| | (0.872) | (0.640) | (1.217) | (0.630) | (0.799) |

This effect was also confirmed when including the time course of the experiment as an influencing factor. The 2-way-ANOVA-test revealed a significant impact of the different experimental groups, the time course (except for FFA) and for the interactions "experimental group — timepoints" on the different parameters (Table 3).

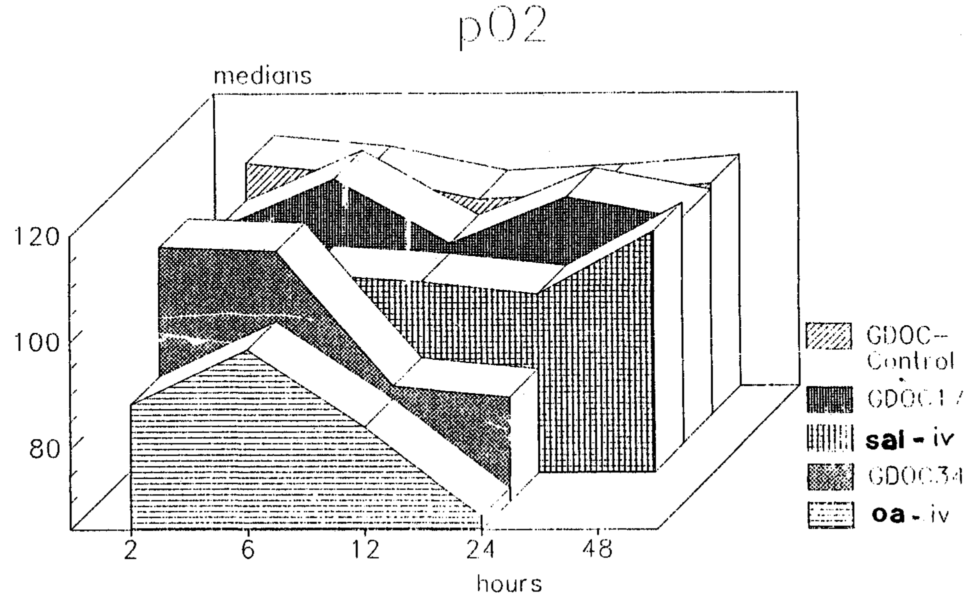

**Figure 3** Levels of p02 (mm Hg); median values + (range)

| | 2 hrs | 6 hrs | 12 hrs | 24 hrs | 48 hrs |
|---|---|---|---|---|---|
| GDOC-34 | 112.5 | 111.8 | 86.4 | 84.3 | — |
| | ( 29.3) | (0 23.2) | ( 15.5) | ( 22.0) | |
| GDOC-17 | 105.3 | 114.6 | 102.5 | 111.1 | 107.2 |
| | ( 29.7) | ( 33.6) | ( 26.9) | ( 47.8) | ( 21.9) |
| GDOC-control | 112.2 | 110.1 | 105.4 | 106.5 | 108.2 |
| | ( 29.2) | ( 20.0) | ( 28.9) | ( 10.2) | ( 22.0) |
| OA i.v. | 88.2 | 98.6 | 84.0 | 67.5 | — |
| | ( 33.7) | ( 21.7) | ( 64.0) | ( 41.4) | |
| SAL i.v. | 94.3 | 102.1 | 100.7 | 98.3 | 110.1 |
| | ( 47.1) | (110.2) | ( 26.4) | ( 20.6) | ( 21.7) |

To analyze the correlation between FFA and the other parameters the Kendall's Tau B-test was performed, results shown in Table 4. According to this, a statistically significant positive correlation of FFA with amylase and lung weight as well as a strongly negative correlation with pO2 was observed. It is noteworthy, that infusion of OA produced beside the expected increase of FFA and the lung injury a significant increase in amylases (compared to SAL) (Figures 1–4; statistics not shown).

### *Morphological Changes*

Gross examination showed pancreatic parenchymatous hemorrhage and pancreatic necrosis in GDOC-34 animals beginning at 2 hours and at all later times; in GDOC-17 animals there was only pancreatic edema until 24 hours and the pancreas appeared normal at 48 hours.

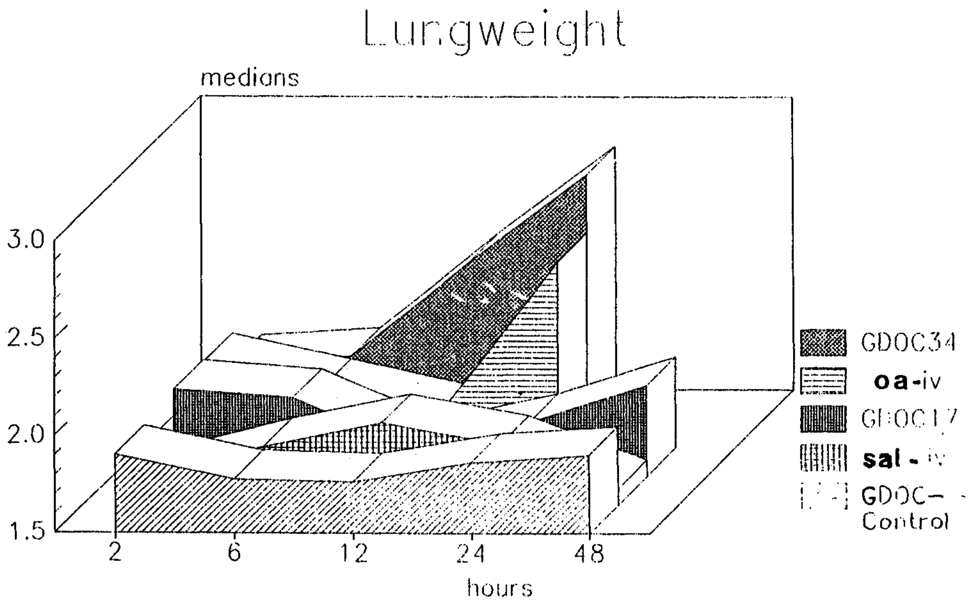

**Figure 4** Lung weight (g); median values + (range)

| | 2 hrs | 6 hrs | 12 hrs | 24 hrs | 48 hrs |
|---|---|---|---|---|---|
| GDOC-34 | 1.780 | 1.820 | 2.300 | 2.745 | — |
| | (0.920) | (0.690) | (1.200) | (1.060) | |
| GDOC-17 | 1.945 | 1.900 | 1.645 | 1.775 | 1.965 |
| | (0.650) | (0.810) | (0.400) | (0.780) | (0.730) |
| GDOC-control | 1.905 | 1.780 | 1.765 | 1.865 | 1.900 |
| | (0.530) | (0.580) | (0.660) | (0.290) | (0.640) |
| OA i.v. | 1.935 | 1.805 | 1.675 | 2.450 | — |
| | (1.040) | (1.000) | (0.560) | (1.200) | |
| SAL i.v. | 1.640 | 1.795 | 1.881 | 1.810 | 1.585 |
| | (1.700) | (.0840) | (0.480) | (0.720) | (0.930) |

In both groups histological examinations of the pancreas showed edema and focal necrosis beginning at 2 hours. Extreme inflammation and hemorrhage were seen in the GDOC-34 group at 12 and 24 hours, but not in GDOC-17 animals. Histological examinations of the pancreas in the OA-group revealed pancreatic edema in all animals after 12 hours and areas of necrosis in 50% of the rats at 24 hours.

Lung examination showed vascular congestion, atelectasis, intra-alveolar edema, interstitial inflammatory infiltrates, and later thickening of the alveolar membrane (Figure 5). Congestion and atelectasis were seen in all animals treated with GDOC-34 and OA but only at 6 hours in the GDOC-17 group. Inflammatory reaction, hemorrhage and thickening of the alveolar membrane occurred in 50% of the OA-animals at 6 hours and 12 hours and in all animals at 24 hours. The same features could be observed in 65% of all GDOC-34 rats at 12 hours and in 80% of

**Table 2** Kruskal-Wallis One Way Analysis of Variance (according to experimental group: *GDOC-34, GDOC-17, OA, GDOC-control, SAL*)

| *Parameters* | *Amylase* | *FFA* | *p02* | *Lung Weight* |
|---|---|---|---|---|
| *p*-values | .0001 | .0001 | .0001 | .011 |

**Table 3** Two Way Analysis of Variance—experimental group by timepoint

| | *Amylase Signif. of F* | *FFA Signif. of F* | *p02 Signif. of F* | *Lung weight Signif. of F* |
|---|---|---|---|---|
| *Source of Variation* | | | | |
| main effects | .0001 | .0001 | .0001 | .0001 |
| experimental group | .0001 | .0001 | .0001 | .0001 |
| timepoint | .0060 | .2600 | .0001 | .0260 |
| 2-way interactions | | | | |
| group-timepoint | .0001 | .0270 | .0680 | .0030 |
| explained variation | .0001 | .0001 | .0001 | .0001 |

**Table 4** Correlation analysis of all parameters (Total experiment, including all groups) (Kendall's Tau B)

| | *FFA* | *Amylase* | *p02* | *Lung weight* |
|---|---|---|---|---|
| *FFA* | | | | |
| Tau b | 1.0000 | .26118 | −.16384 | .14060 |
| p-value | | .00001 | .00220 | .00750 |
| *AMYLASE* | | | | |
| Tau b | .26118 | 1.00000 | −.17668 | .15540 |
| p-value | .00001 | | .00110 | .00360 |
| *p02* | | | | |
| Tau b | −.16384 | −.17668 | 1.00000 | −.17604 |
| p-value | .00220 | .00110 | | .00120 |
| *LUNG WGHT* | | | | |
| Tau b | .14060 | .15540 | −.17604 | 1.00000 |
| p-value | .00750 | .00360 | .00120 | |

all animals in this group at 24 hours. In contrast, only one rat in the GDOC-17 group developed pulmonary hemorrhage at 24 hours, and no alveolar wall thickening was found in the GDOC-17 group at any time.

### DISCUSSION

In 20–60% of patients acute pancreatitis is associated with lung injury, which may progress to alterations consistent with ARDS[1–6]. We have examined the occurrence

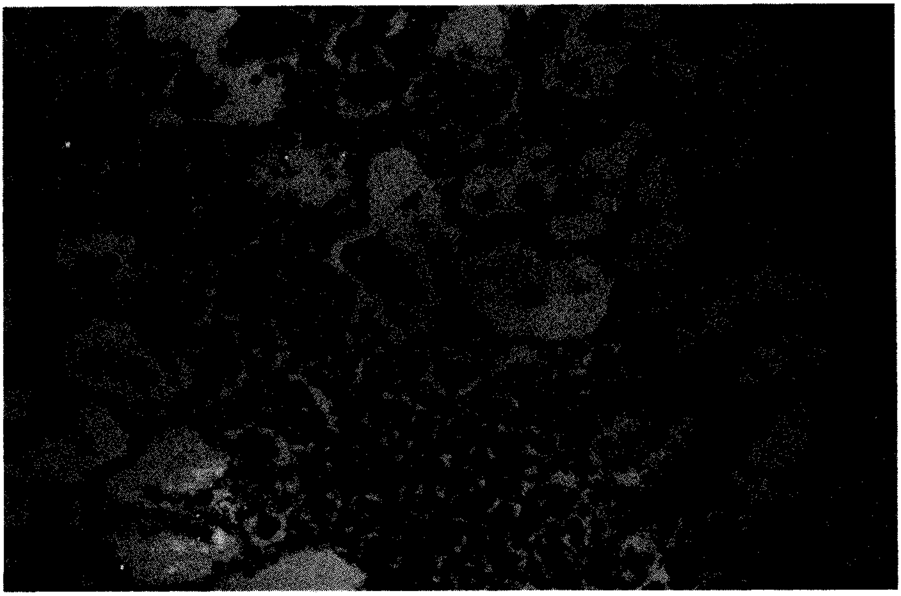

**Figure 5** Marked inflammatory infiltrates with thickening of the alveolar membrane and intravascular congestion. (*See colour plate at back of issue*).

and time course of pulmonary changes in an experimental model of acute pancreatitis as well as after i.v. infusion of oleic acid. We further correlated serum levels of free fatty acids with the evolution of the lung injury, as several authors have stated an important role of free fatty acids in the pathogenesis of pancreatitis and subsequent lung injury[7,9,10,15]. By use of glycodeoxycholic acid in two different concentrations (34 mmol and 17 mmol) we were able to create pancreatitis of two different levels of severity and degrees of pancreatitis associated pulmonary injury.

Application of 34 mmol GDOC produced fulminant acute pancreatitis with early edema and focal necrosis, progressing to extreme inflammation and hemorrhage by 12 hours. These animals had an increase in lung weight and continuously rising amylase levels, combined with a decrease in arterial pO2, and no animal survived longer than 30 hours. The FFA levels in this group at 24 hours were significantly higher than in controls. Pathologic lung alterations (congestion of lung capillaries, inflammatory reaction, interalveolar edema) were found to precede the appearance of clinically manifest hypoxia, as has been reported by other investigators[16]. The observations in the GDOC-34 group were identical to those seen in rats with i.v. infusion of oleic acid, a substance which belongs to the group of "free fatty acids". The intravenous application of oleic acid has been previously used as an experimental model to create ARDS-like severe lung injury[9,17]. It has been repeatedly demonstrated that the infusion of oleic acid leads to a damage of the alveolary-capillary membrane and a subsequent pulmonary injury comparable to ARDS in humans[9,17,18].

Unexpectedly, an increase of levels of amylases associated with pancreatic edema and focal pancreatic necroses in three of six rats at 24 hours in the OA-group was observed.

In contrast to this, the animals treated with GDOC-17 had much milder consequences. No animal in this group died of pancreatitis. Although they developed edema and focal necrosis by histological examination within two hours, these changes were not progressive. Amylase levels rose initially but fell steadily back. PO2, lung weight and FFA never differed significantly from control values. All animals had clinical evidence of recovery as manifested by resumption of food intake and excretion. Morphologic lung changes were essentially confined to congestion and atelectasis: one animal developed pulmonary hemorrhage at 24 hours, but alveolar thickening was observed in no instance in the GDOC-17 group.

These findings show that we were able to create two different levels of severity of acute pancreatitis. There appears within these different subsets to be concordance between severity of pancreatitis, the degree of pulmonary dysfunction and the morphologic changes in the lungs.

In the same manner, free fatty acids elevations also correlated with severe lung injury confirming previous observations about the possible cause-and-effect relationship of FFA and acute pancreatitis: Schmidt and Lankisch and others have suggested that parenchymal lesions seen during acute pancreatitis in rats are due to the detergent effect of released non-esterified free fatty acids[19,20]. They performed comparative studies in patients who died of acute pancreatitis and concluded that lipase-induced release of cytotoxic free fatty acids caused propagation and deterioration of the disease[19]. Release of FFA during acute pancreatitis may be triggered by lipolytic enzymes, in particular phospholipase A2[21,22].

Other experimental studies in the isolated canine pancreas have shown that perfusion of the pancreas with oleic acid produces severe tissue damage with acute necrotising pancreatitis[23]. Similar to these observation, i.v. infusion of oleic acid produced an increase of levels of amylases and alterations of the pancreatic parenchyma in our experiment.

It has been reported that persistence of acute pancreatitis is associated with elevated phospholipase A2 levels[22], and that phospholipase A2 activity is also increased in the lung in experimental acute pancreatitis[24]. Recently, Büchler *et al.* have shown in man that elevated serum levels of phospholipase A2 are particularly associated with pancreatic necrosis and pulmonary failure[25]. The association we have noted between severe pancreatitis, lung injury and elevation of serum FFA may be consistent with these clinical observations since it is well known that the activity of phospholipases leads to the liberation of FFA and lysolecithin. In a recent paper from the above group 20 patients with acute edematous pancreatitis differed significantly from an equal number of patients who suffered from acute necrotizing pancreatitis regarding their FFA levels at the time of admission as well as during the further course of their disease[7].

Although these clinical observations are in concordance with the results of our animal experiment a final conclusive statement regarding the causal relationship of FFA levels and severity of pancreatitis cannot be made. It must be taken into account that the increase of levels of FFA might be only a nonspecific expression of stress caused by a severe disease.

*Acknowledgements*

This study was performed during a research fellowship at the Department of Surgery at the Massachusetts General Hospital, Boston, Ma., which was partly supported by the Austro-American Educational Commission (Fulbright Commission) and the Austrian Society of Surgery.

The authors wish to express their thanks to Univ. Prof. Dr G. Schlag, head of the Ludwig Boltzmann Institute for Clinical and Experimental Traumatology, Vienna, Austria for his highly constructive comments and suggestions regarding this presentation.

Furthermore, the authors have to thank Dr Carolyn Compton, Associate Professor of Pathology, Harvard University, Boston, Ma., for her thoroughful examination of the histologic specimens of the experiment.

### *References*

1. Cameron, A.G. (1975) Acute respiratory failure in acute pancreatitis. *Anaest. Inten. Care*, **3**, 244–260
2. Hayes, M.F., Rosenbaum, R.W., Ziebelman, M. and Matsumotu, T. (1974) Adult respiratory distress syndrome in association with adult pancreatitis. *Am. J. Surg.*, **127**, 314–318
3. Murphy, D., Imrie, C.W., Pack, A., Davidson, J.F. and Blumgart, L.H. (1976) The mechanism of acute respiratory insufficiency in acute pancreatitis. *Br. J. Surg.*, **63**, 669–673
4. Gliedman, M.L., Bolooki, H. and Rosen, R.G. (1970) Acute pancreatitis. *Curr. Probl. Surg.*, **15**, 432–437
5. Stömmer, P. (19 ) Lungenschäden durch akute tryptische Pankreatitis. *Dtsch. med. Wschr.*, **109**, 454–458
6. Hollender, C.F., Lehnert, P. and Wanke, M. (1983) *Akute Pankreatitis, eine interdisziplinäre Synopsis*. München. Urban und Schwarzenberg
7. Domschke, S., Malfertheiner, P., Büchler, M. and Domschke, W. (1990) Prolonged increase in serum free fatty acids in acute necrotizing pancreatitis compared to oedematous pancreatitis. In: Abstracts of the World Congresses of Gastroenterology. Sydney 1990 Abingdon: The Medicine Group (UK) Ltd., p. 354
8. Warshaw, A.L., Lesser, P.B., Rie, M. and Cullen, D.I. (1975) Pathogenesis of pulmonary edema in acute pancreatitis. *Ann. Surg.*, **12**, 505–511
9. Kimura, T., Toung, S.K. and Margolis, S. (1979) Respiratory failure in acute pancreatitis: a possible role for triglycerides. *Ann. Surg.*, **189**, 509–513
10. Kimura, T., Toung, J.K. and Margolis, S. (1980) Respiratory failure in acute pancreatitis: the role of free fatty acids. *Surgery*, **87**, 509–514
11. Terry, T.R., Grant, D.A.W. and Hermon-Taylor, J. (1987) Intraduct enterokinase is lethal in rats with experimental bile-salt pancreatitis. *Br. J. Surg.*, **74**, 40–43
12. Tarpila, E., Nyström, P.O., Franzen, L., Lilija, I. and Ihse, I. (1988) Acute experimental suppurative pancreatitis in the rat. *Acta Chir. Scand.*, **154**, 379–383
13. Bernfeld, P. (1951) Enzymes of starch degradation and synthesis. *Adv. Enzym.*, **12**, 379–391
14. Mulder, C., Schouten, J.A., Popp-Snijders, C. (1983) Determination of free fatty acids: a comparative study of the enzymatic versus the gas chromatographic and the colorimetric method. *J. Clin. Chem. Clin. Biochem.*, **21**, 823–827
15. Shaw, W. (1985) Possible role of lysolecithins and nonesterified fatty acids in the pathogenesis of Reye's syndrome, sudden infant death syndrome, acute pancreatitis and diabetic ketoacidosis. *Chir. Chem.*, **31**(7), 1109–1115
16. Burnweit, C.A. and Horton, I.W. (1988) Extravascular lung water as an indicator of pulmonary dysfunction in acute hemorrhagic pancreatitis. *Ann. Surg.*, **207**, 33–38
17. Dickey, B.F., Thrall, R., McCormick, J.R. and Ward, P.A. (1981) Oleic-acid-induced injury in the rat. *Am. J. Pathol.*, **103**, 376–383
18. Ashbaugh, D.G. and Uzawa, T. (1968) Respiratory and hemodynamic changes after injection of free fatty acids. *J. Surg. Res.*, **8**, 417–422
19. Schmidt, H. and Lankisch, P. (1978) Fat necrosis-a cause of pancreatic parenchymal necrosis. *Digestion*, **17**, 84–89
20. Schmitz-Moormann, P. and Boger, A. (1981) Tissue damage by fatty acids released by lipolysis. Contribution to the pathogenesis of acute pancreatitis. *Pathol. Res. Pract.*, **171**, 303–307
21. Aho, J., Nevelainen, T.I., Lindenberg, R.L.P. and Aho, A.I. (1984) Experimental pancreatitis in the rat: the role of phospholipase-A in sodium taurocholate induced acute haemorrhagic pancreatitis. *Scand. J. Gastroenterol.*, **15**, 1027–1035

22. Hölbling, N., El-Kalak, H., Georgopoulos, A., Stilianou, L. and Hacker, G. (1985) Phospholipase-A1 and -A2 in experimental acute pancreatitis in rats: *Res. Exp. Med.*, **131**, 320–324
23. Sarr, M.G., Bulkley, G.B. and Cameron, I.L. (1985) The role of leukocytes in the production of oxygen-derived free radicals in acute experimental pancreatitis. *Surgery*, **101**(3), 292–296
24. Das, S.K., Scott, M.T. and McCuiston, S. (1987) Effects of experimental acute pancreatitis in dog on metabolism of lung surfactant phosphatidylcholine. *Biochem. Biophys. Res. Comm.*, **145**(1), 612–619
25. Büchler, M., Malfertheiner, P., Schädlich, H., Nevelainen, T.J., Friess, H. and Beger, H.G. (1989) Role of phospholipase A2 in human acute pancreatitis. *Gastroenterology*, **97**, 1521–1528

(*Accepted by S. Bengmark 12 April 1992*)

